# Differential Modulation of the DR5–JNK–PD-L1 Signaling Axis by TRiC/CCT Subunits CCT7 and CCT2 Shapes Tumor Immune Evasion in Lung Adenocarcinoma

**DOI:** 10.3390/cells15171613

**Published:** 2026-09-04

**Authors:** Hsin-Wei Jen, Hsiang-Ling Ho, Teh-Ying Chou

**Affiliations:** 1 Translational Medicine Center, Department of Research, Shin Kong Wu Ho-Su Memorial Hospital, Taipei 111, Taiwan; 2 Department of Pathology and Laboratory Medicine, Taipei Veterans General Hospital, Taipei 112, Taiwan; 3 Department of Biotechnology and Laboratory Science in Medicine, National Yang Ming Chiao Tung University, Taipei 112, Taiwan; 4 Department of Pathology and Laboratory Medicine, Shin Kong Wu Ho-Su Memorial Hospital, Taipei 111, Taiwan; 5 Graduate Institute of Clinical Medicine, College of Medicine, Taipei Medical University, Taipei 110, Taiwan

**Keywords:** CCT7, CCT2, PD-L1, DR5, JNK, c-Jun

## Abstract

**Highlights:**

**What are the main findings?**

- CCT7 and CCT2 exert opposing effects on PD-L1 expression and T-cell activation in lung adenocarcinoma.
- CCT7 depletion activates the DR5–MKK4–JNK–c-Jun signaling cascade, leading to PD-L1 upregulation and T-cell suppression, whereas CCT2 depletion produces the opposite effects.

**What are the implications of the main findings?**

- These findings reveal distinct, subunit-specific roles of the TRiC/CCT complex in tumor immune regulation.
- Understanding these divergent roles may provide new opportunities for developing more precise cancer immunotherapy strategies.

**Abstract:**

While the chaperonin-containing TCP-1 (CCT) complex is essential for proteostasis, the distinct roles of individual subunits in tumor immune regulation remain unclear. Here, we identify CCT7 as a previously unrecognized regulator of immune evasion in lung adenocarcinoma (LUAD). Integrative analyses of TCGA and GEO cohorts revealed that CCT7 is markedly upregulated in LUAD and is associated with poor patient prognosis. Functional studies demonstrated that CCT7 knockdown inhibited tumor cell proliferation and migration and enhanced cisplatin-induced apoptosis, yet paradoxically impaired T-cell activation. Mechanistically, transcriptomic and biochemical analyses revealed that CCT7 depletion activated the DR5–MKK4–JNK–c-Jun signaling cascade, resulting in the transcriptional upregulation of PD-L1. Disruption of DR5 or JNK signaling effectively abrogated PD-L1 induction. In contrast, CCT2 depletion exerted the opposite effect by suppressing the DR5–JNK–c-Jun–PD-L1 signaling axis and enhancing T-cell activation. Collectively, these findings reveal unexpected functional divergence among TRiC/CCT subunits and identify the CCT7–DR5–JNK–c-Jun signaling axis as a previously unrecognized mechanism regulating PD-L1-mediated immune evasion, highlighting the potential therapeutic relevance of this signaling axis in LUAD.

## 1. Introduction

Non-small cell lung cancer (NSCLC) is the most common histological subtype of lung cancer, accounting for approximately 85% of all cases worldwide. Despite substantial advances in surgery, chemotherapy, radiotherapy, targeted therapy, and immunotherapy, the prognosis for patients with advanced NSCLC remains poor, with a 5-year survival rate below 25% [1]. One major obstacle is late-stage diagnosis, as many patients present with locally advanced or metastatic disease at the time of diagnosis, rendering curative surgical resection infeasible [2]. Even for early-stage tumors treated surgically, recurrence rates remain high due to minimal residual disease and micro-metastasis [3].

In patients with lung cancer, metastasis is the main cause of mortality [4]. Although many chemotherapeutic drugs have been specifically designed to inhibit the expression of oncoproteins, the response rate of patients with metastasis remains low [5]. Immune checkpoint inhibitors (ICIs) are often used in the treatment of patients with advanced stage disease [6]. According to clinical trials, ICI therapy is more effective than chemotherapy at increasing the survival and response rates of patients with cancer. Immune checkpoint blockade (ICB) targeting the PD-1/PD-L1 axis has fundamentally transformed the management of lung adenocarcinoma (LUAD), providing durable survival benefits in biomarker-selected patients [7]. Although ICI therapy increases the survival rate of patients, approximately 50% of PD-L1-positive patients do not respond to ICIs [5]; only a subset of patients achieves long-term responses, and predictive biomarkers remain imperfect [8].

The eukaryotic chaperonin-containing TCP-1 (CCT), also known as the TRiC complex (TCP-1 Ring Complex), is a type II cytosolic chaperonin essential for the folding of ~10% of the eukaryotic proteome, including cytoskeletal proteins such as actin and tubulin, and diverse signaling molecules [9]. Structurally, TRiC/CCT consists of eight distinct subunits (CCT1–CCT8), each contributing unique substrate-binding specificities, thereby enabling the complex to regulate protein homeostasis in a highly selective manner [10]. In cancer, TRiC/CCT has emerged as more than a housekeeping chaperone. Dysregulation of its subunits has been linked to malignant transformation, tumor progression, and therapy resistance. For example, overexpression of CCT2, CCT3, and CCT7 has been reported in breast, hepatocellular, and lung cancers, and these alterations correlate with a poor prognosis [11,12,13]. Mechanistically, TRiC/CCT facilitates the folding of oncogenic client proteins including STAT1, STAT3, KRAS, and components of the Wnt/β-catenin and PI3K/AKT/mTOR pathways [14,15,16,17].

Mammalian T lymphocytes rely on antigen-presenting cells to form immunological synapses (ISs), where centrosome polarization and cytoskeletal remodeling are essential for full activation [18,19]. Recent work shows that TRiC/CCT is critical for adaptive immunity by regulating T-cell polarity and metabolism. TRiC/CCT promotes protein folding and supports tubulin assembly into microtubules, thereby sustaining cytoskeletal organization and metabolic reprogramming during IS formation [20]. Bioinformatic studies using TCGA and CCLE datasets indicate that elevated CCT2 expression correlates with reduced immune cell infiltration and altered tumor mutation profiles [21]. In human CD4^+^ T cells, transient CCT knockdown reduces calcium flux and NFAT activation by impairing Orai1 internalization at the plasma membrane, linking TRiC/CCT to calcium signaling and transcriptional control [22]. PD-L1 profoundly shapes the balance of Th1 and Th2 immune responses within the tumor microenvironment. Engagement of PD-L1 with PD-1 on CD4^+^ T cells suppresses T-cell receptor (TCR) signaling, leading to reduced secretion of Th1-associated cytokines such as IFN-γ and IL-2 and impairing effector differentiation and cytotoxicity [23]. This inhibition of Th1 activation diminishes the recruitment of additional Th1 cells, thereby attenuating cell-mediated antitumor immunity [24]. An imbalance favoring Th2 polarization has been associated with impaired antitumor immunity and poor clinical outcomes in several cancers, including lung cancer [25]. Loss of CCT8 leads to defective thymocyte maturation and impaired T-cell activation. Moreover, CCT8 deficiency alters Th2 differentiation and paradoxically sustains IFN-γ expression, resulting in ineffective immune protection [26]. Recently, CCT5 has been shown to promote colorectal cancer progression by enhancing asparagine biosynthesis and PD-L1 expression, thereby impairing CD8^+^ T-cell responses [27]. These findings indicate that TRiC/CCT governs T-cell activation, polarity, and lineage commitment. However, how the TRiC/CCT complex modulates the immune responsiveness of cancer cells remains to be further elucidated.

Death receptor 5 (DR5; TNFRSF10B) is a member of the tumor necrosis factor receptor superfamily and is best known for mediating TRAIL-induced apoptotic signaling. In addition to its canonical apoptotic function, DR5 can also engage non-apoptotic signaling pathways, including MAPK signaling. JNK is activated through phosphorylation at Thr183/Tyr185 by upstream MAP2Ks, including MKK4, and subsequently phosphorylates c-Jun, thereby enhancing AP-1 transcriptional activity. Emerging evidence suggests that JNK/c-Jun signaling can also contribute to the regulation of PD-L1 expression and tumor immune responses [28,29].

In this study, we demonstrate that CCT7 functions as a previously unrecognized regulator of tumor–immune interactions in lung adenocarcinoma. Knockdown of CCT7 enriches immune and inflammatory signaling programs, impairs Jurkat T-cell responses, and markedly increases PD-L1 expression in lung cancer cells. Mechanistically, CCT7 silencing activates the DR5/MKK4/JNK/c-Jun signaling axis, leading to transcriptional upregulation of PD-L1. In contrast, knockdown of CCT2 exerts the opposite effect on the DR5/JNK/c-Jun/PD-L1 pathway, highlighting functional divergence among TRiC/CCT subunits. Together, these findings identify a chaperonin-dependent signaling mechanism that regulates immune checkpoint responses in lung cancer.

## 2. Materials and Methods

### 2.1. Cell Culture and Reagents

The human lung adenocarcinoma cell line CL1-5 was kindly provided by Professor Pan-Chyr Yang (National Taiwan University, Taipei, Taiwan). H1299, H1975 and Jurkat T cells were obtained from the American Type Culture Collection (ATCC, Manassas, VA, USA). Cells were cultured in RPMI-1640 medium (Gibco, Grand Island, NY, USA; 11875093) supplemented with 10% fetal bovine serum (FBS) (Corning, Corning, NY, USA; 35-010-CV), 100 U/mL penicillin, and 100 μg/mL streptomycin (Gibco, Grand Island, NY, USA; 15140-122). All cell lines were maintained at 37 °C in a humidified incubator with 5% CO_2_. The JNK inhibitor SP600125 was purchased from MedChemExpress (MCE, Monmouth Junction, NJ, USA; HY-12041).

### 2.2. Plasmid and siRNA Transfection

pcDNA3-TRAIL-R2 (DR5) (plasmid #61383) was purchased from Addgene (Watertown, MA, USA), and siRNAs targeting the indicated genes and a non-targeting control siRNA were synthesized by Bioneer (Daejeon, Republic of Korea). The siRNA sequences used were as follows: siCCT7-1: CCACACAGUUGAGGAUUAUdTdT; siCCT7-2: CCAUCAUUCUGGAGCCAAAdTdT; siPD-L1: CCAGCACACUGAGAAUCAAdTdT; sic-Jun: AAGAAGACAUCAUCCGGAAUA; siCCT2: GCACAACAUUAUCCUCAAAdTdT; siDR5: AUCAGCAUCGUGUACAAGGUGUCCCdTdT; control siRNA (siCtrl): UUCUCCGAACGUGUCACGUdTdT. siCCT7 represents a mixture of two siRNAs targeting CCT7 (siCCT7-1 and siCCT7-2). Cells were transiently transfected with small interfering RNAs (siRNAs) using Lipofectamine™ RNAiMAX (Thermo Fisher Scientific, Waltham, MA, USA; 13778150) according to the manufacturer’s instructions. siRNA–RNAiMAX complexes were prepared in Opti-MEM^®^ Reduced Serum Medium (Gibco™, 31985070) and incubated at room temperature for 10 min before being added to the cells. After transfection, cells were maintained in complete growth medium and harvested at the indicated time points for subsequent analyses.

### 2.3. Apoptosis Assay

Apoptosis was assessed using the Annexin V Apoptosis Detection Kit I (BD Biosciences, Franklin Lakes, NJ, USA; Cat# 556547) according to the manufacturer’s instructions. Briefly, H1299 and CL1-5 lung cancer cells were transfected with control siCtrl or siCCT7. After 48 h of siRNA transfection, cells were treated with cisplatin (30 μM) for an additional 24 h to induce apoptosis. Cells were then stained with Annexin V–FITC and propidium iodide (PI) for 15 min at room temperature in the dark. After staining, samples were analyzed by flow cytometry.

### 2.4. Cell Viability Assay

Cell viability was assessed using the Cell Counting Kit-8 (GlpBio, Montclair, CA, USA; GK10001) according to the manufacturer’s instructions. Briefly, lung cancer cells were seeded into 96-well plates at a density of 5 × 10^3^ cells per well and then transfected with control siRNA or CCT7 siRNA and cultured for 48 h. At the indicated time points, 10 μL of CCK-8 solution was added to each well containing 90 μL of culture medium. The plates were then incubated at 37 °C for 1 h. The absorbance was measured at 450 nm using a microplate reader.

### 2.5. Transwell Migration Assay

The migration capacity of cells with CCT7 knockdown was evaluated using 24-well Transwell chambers with 8 μm pore-size polycarbonate membranes (Corning, NY, USA). After CCT7 siRNA or control siRNA treatment for 48 h, cells were harvested and resuspended in serum-free medium. A total of 1~5 × 10^4^ cells in 200 μL of serum-free medium were seeded into the upper chamber, while 700 μL of complete medium containing 10% FBS was added to the lower chamber to serve as a chemoattractant. Following incubation at 37 °C for 24 h, cells remaining on the upper surface of the membrane were gently removed using a cotton swab. The migrated cells on the lower surface were fixed with 4% paraformaldehyde for 15 min and subsequently stained with 0.1% crystal violet for 10 min. The number of migrated cells was quantified by counting five randomly selected fields per well under an inverted light microscope. All experiments were performed in triplicate, and the results were expressed as the mean number of cells per field.

### 2.6. Wound Healing Assay

Cells were transfected with CCT7 siRNA or control siRNA for 48 h in 6-well plates and cultured until they reached approximately 90~95% confluence. Afterward, a sterile 200 μL pipette tip was used to create a straight scratch in the cell monolayer. The wells were washed twice with PBS to remove cellular debris and then replenished with medium containing 1% FBS to minimize the influence of cell proliferation. Images of the wound area were captured at 0 and 48 h using a microscope. The width of the wound was measured using ImageJ software version 1.54 (National Institutes of Health, Bethesda, MD, USA).

### 2.7. Western Blotting

Total cellular lysates were collected using RIPA buffer (Thermo Fisher Scientific, 89900) containing a protease inhibitor cocktail (Roche, Basel, Switzerland; cOmplete) and a phosphatase inhibitor cocktail (Roche, Basel, Switzerland; PhosSTOP). Equal amounts of protein lysates (20 μg) were mixed with sample loading buffer and boiled at 100 °C for 10 min. All protein lysates were loaded into 10%~12% SDS-PAGE gels and transferred onto a 0.45 μm PVDF membrane. The PVDF membranes were blocked with 5% non-fat milk for 1 h at room temperature and then incubated with primary antibodies overnight at 4 °C. Following three washes with PBST, the membranes were incubated with HRP-conjugated secondary antibodies for 1 h at room temperature. Protein bands were visualized using an enhanced chemiluminescence (ECL) detection system. The primary antibodies used were as follows: anti-CCT7 (Santa Cruz Biotechnology, Dallas, TX, USA; sc-271951), anti-CCT2 (Abcam, Cambridge, UK; ab92746), anti-GAPDH (Santa Cruz Biotechnology, Dallas, TX, USA; sc-47724), anti-PD-L1 (GeneTex, Irvine, CA, USA; GTX104763), anti-PARP1 (GeneTex, Irvine, CA, USA; GTX100573), anti-c-Jun (Cell Signaling, Danvers, MA, USA; #9165), anti-p-c-Jun (Biotechnology, Dallas, TX, USA; sc-822), anti-p-JNK (Cell Signaling, Danvers, MA, USA; #4668), anti-JNK (Proteintech, Rosemont, IL, USA; 10023-1-AP), anti-pMKK4 (Cell Signaling, Danvers, MA, USA; #4514), anti-MKK4 (Cell Signaling, Danvers, MA, USA; #9152), anti-DR5 (Cell Signaling, Danvers, MA, USA; #8074), anti-BCL2 (Proteintech, Rosemont, IL, USA; 12789-1-AP), anti-Caspase-3 (Cell Signaling, Danvers, MA, USA; #9662).

### 2.8. Human Phospho-Kinase Array

Phosphorylation profiles of multiple kinases were analyzed using a Human Phospho-Kinase Array Kit (R&D Systems, Minneapolis, MN, USA; ARY003C) according to the manufacturer’s instructions. H1975 cells were transfected with either siCtrl or siCCT7 for 48 h. Subsequently, whole-cell lysates were harvested, and equal amounts of protein were incubated overnight with the array membranes. Captured phospho-proteins were detected using a cocktail of biotinylated antibodies and streptavidin-HRP, followed by chemiluminescent visualization.

### 2.9. IL-2 ELISA Assay

Prior to co-culture, 48-well plates were pre-coated with anti-CD3 antibody (Biolegend, San Diego, CA, USA; 317347) at 5 μg/mL in PBS at 4 °C overnight, followed by three washes with PBS to remove unbound antibodies. For T-cell activation, Jurkat cells were stimulated with plate-bound anti-CD3 antibody and soluble anti-CD28 antibody for 24 h. Lung cancer cells were transfected with target siRNA or control siRNA for 24 h in 24-well plates. Afterward, activated Jurkat cells (effector cells) were added to the target cells at an effector-to-target (E:T) ratio of 5:1 in medium supplemented with soluble anti-CD28 antibody (Biolegend, 302943) at 2 μg/mL to provide costimulatory signals. After 48 h of co-culture, the supernatants were collected and centrifuged at 1000× *g* for 10 min at 4 °C to remove cellular debris. The concentration of secreted IL-2 was quantified using a human IL-2 ELISA kit (Invitrogen, Waltham, MA, USA; BMS221INST) following the manufacturer’s protocol. Absorbance was measured at 450 nm, and IL-2 levels were calculated based on a standard curve. All assays were performed in triplicate.

### 2.10. Flow Cytometry

For cell surface PD-L1 protein detection, CL1-5, H1299, and H1975 lung cancer cells were transfected with siCtrl, siCCT7, or siCCT2 and incubated for 48 h. Cells were then harvested, washed with cold phosphate-buffered saline (PBS), and resuspended in staining buffer (PBS containing 1% bovine serum albumin). Cells were incubated with a primary antibody against PD-L1 (Abcam, ab205921) for 30 min at 4 °C, followed by washing and incubation with an Alexa Fluor 488-conjugated secondary antibody (Invitrogen, A11034) for 30 min at 4 °C in the dark. After staining, cells were washed with PBS and resuspended in staining buffer for analysis.

For the Jurkat T-cell activation assay by CD69 flow cytometry, H1299 lung cancer cells were transfected with siCtrl or siCCT7 and cultured for 24 h. Activated Jurkat T cells (CD3/CD28 stimulation) were then added to the H1299 cells and co-cultured for 24 h. After co-culture, Jurkat cells were collected and washed with cold phosphate-buffered saline (PBS). Cells were stained with anti-human CD69 antibody (BioLegend, Cat# 310911) for 30 min at 4 °C in the dark. After washing, cells were resuspended in PBS and analyzed by flow cytometry.

### 2.11. Quantitative Reverse Transcription PCR

Total RNA was extracted from CL1-5, H1299 and H1975 lung cancer cells transfected with control siRNA (siCtrl) or CCT7-targeting siRNA (siCCT7) using TRIzol reagent according to the manufacturer’s instructions (ZYMO research, Irvine, CA, USA). RNA was reverse transcribed to complementary DNA (cDNA) using the Maxima H Minus First Strand cDNA Synthesis Kit (Thermo Fisher Scientific). The mRNA levels were analyzed using a SYBR Green PCR Master Mix (Thermo Fisher Scientific) and a StepOnePlus™ System (Thermo Fisher Scientific, Waltham, MA, USA). The relative mRNA expression levels were calculated using the 2^−ΔΔCt^ method, with GAPDH serving as the internal reference control. The primer sequences were as follows: PD-L1 forward 5′-GGACAAGCAGTGACCATCAAG-3′ and reverse 5′-CCCAGAATTACCAAGTGAGTCCT-3′, GAPDH forward 5′-TGGGCTACACTGAGCACCAG-3′ and reverse 5′-CAGCGTCAAAGGTGGAGGAG-3′, and DR5 forward 5′-CCAGCAAATGAAGGTGATCC-3′ and reverse 5′-GCACCAAGTCTGCAAAGTCA-3′.

### 2.12. Transcriptomic Analysis

In the CL1-5, H1299 and H1975 cell lines, differentially expressed genes (DEGs) between siCCT7- and siCtrl-treated cells were identified using a statistical model, with genes meeting the criteria of |log_2_FC| ≥ 0.58 and an adjusted false discovery rate (FDR) < 0.05 considered significant. To characterize the functional relevance of CCT7-regulated transcriptional changes, gene set enrichment analysis (GSEA) was performed using the Hallmark and Gene Ontology Biological Process (GOBP) gene sets, and the top 10 enrichment pathways were displayed. Enrichment scores were calculated based on ranked gene lists, and statistical significance was determined using FDR-adjusted q-values. Gene expression data from GSE2514 and GSE32863 were obtained from the Gene Expression Omnibus (GEO) database. The datasets include gene expression profiles of lung cancer and normal lung tissues and were used to evaluate CCT7 expression patterns.

### 2.13. Proteomic Analysis

To investigate changes in the expression of CCT complex subunits following CCT7 knockdown, proteomic analysis was performed in H1975 cells. Cells were transfected with control siRNA (siCtrl) or CCT7-targeting siRNA (siCCT7) and cultured for 48 h. Total cellular proteins were extracted using lysis buffer, and protein concentrations were determined prior to analysis. Protein samples were subjected to tryptic digestion and analyzed by liquid chromatography–tandem mass spectrometry (LC–MS/MS). The resulting spectra were searched against the human protein database, and relative protein abundance was quantified based on peptide intensity. The expression levels of the eight CCT subunits (CCT1–CCT8) were extracted from the proteomic dataset and compared between siCtrl and siCCT7 conditions.

### 2.14. Clinical Survival and Gene Correlation Analysis

Survival analyses were performed using the GEPIA2 web server (http://gepia2.cancer-pku.cn/ (accessed on 1 June 2024)), which integrates RNA sequencing data from The Cancer Genome Atlas (TCGA) project. Kaplan–Meier survival analyses were performed to evaluate the association between CCT family gene expression and patient survival in lung adenocarcinoma (LUAD). Patients were divided into high- and low-expression groups using a median cutoff (50%). Survival differences were evaluated using the log-rank test, and *p* values < 0.05 were considered statistically significant. Pairwise gene expression correlations were calculated using the Spearman correlation method, and the correlation coefficient (R) and corresponding *p*-values were obtained from the GEPIA2 platform. Scatter plots were generated to visualize the relationships between PD-L1 and the indicated genes.

### 2.15. Statistical Analysis

All quantitative data are presented as mean ± SD from three to five independent experiments. Statistical analyses were performed using GraphPad Prism 9 (GraphPad Software, San Diego, CA, USA). Differences between two groups were evaluated using a two-tailed Student’s *t*-test. A *p* value < 0.05 was considered statistically significant.

## 3. Results

### 3.1. CCT7 Knockdown Suppresses Cell Proliferation and Migration and Induces Apoptosis in Lung Adenocarcinoma Cells

**Figure 1:**
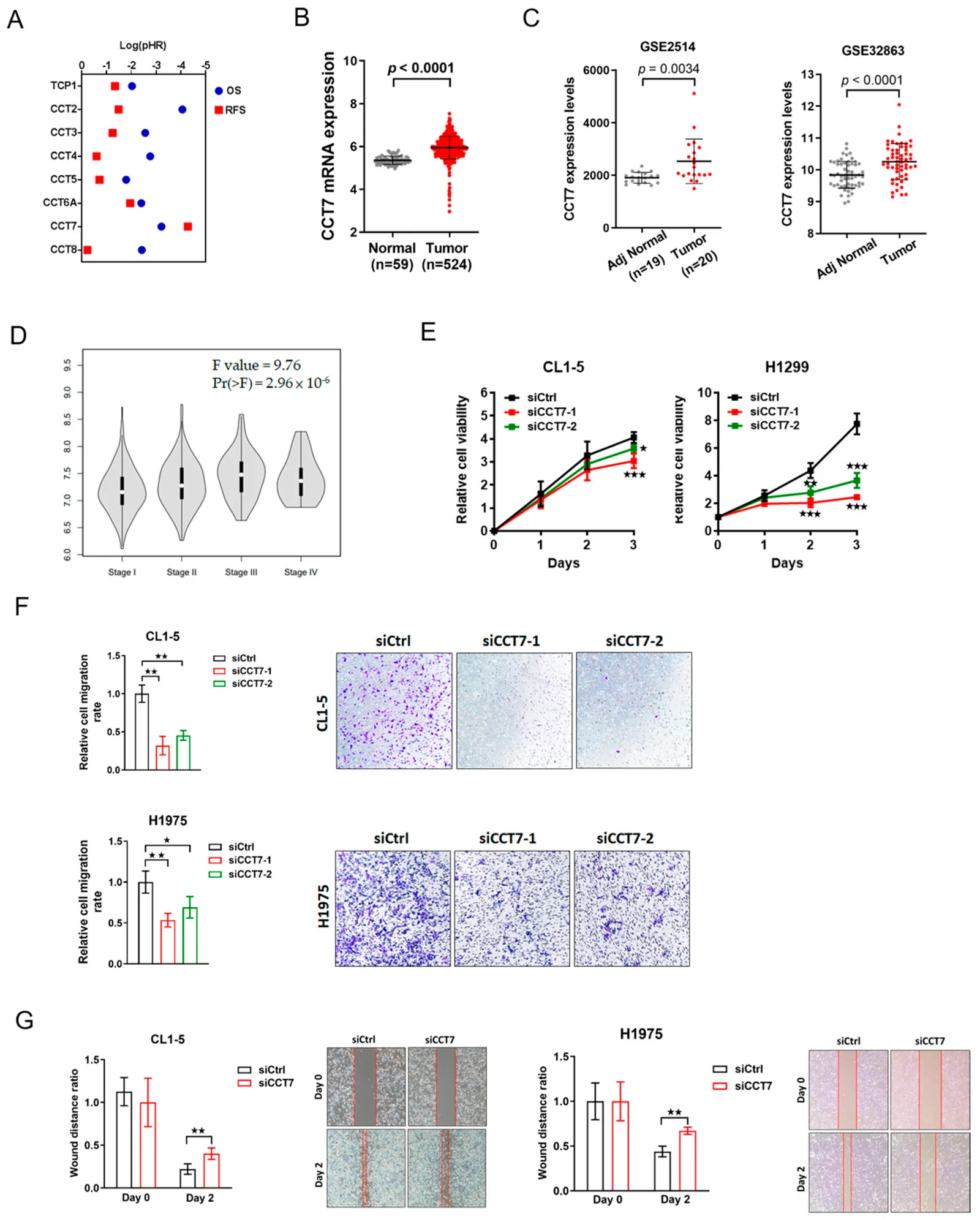
Effects of CCT7 Silencing in Lung Cancer Cells. (**A**) Dot plot showing the association between expression levels of individual TRiC/CCT subunits and overall survival (OS) and relapse-free survival (RFS) in lung adenocarcinoma cohorts. (**B**) CCT7 expression levels in normal lung tissues and lung adenocarcinoma tissues from the TCGA LUAD dataset. (**C**) CCT7 expression in lung adenocarcinoma specimens and matched adjacent normal tissues in two independent GEO cohorts (GSE2514 and GSE32863). (**D**) Violin plots depicting CCT7 expression levels across different tumor stages in lung adenocarcinoma from the TCGA LUAD dataset. (**E**) Cell proliferation assays of CL1-5 and H1299 lung cancer cells following siRNA-mediated knockdown of CCT7, measured over the indicated time points (*n* = 3). (**F**) Transwell migration assays of CL1-5 and H1975 cells transfected with control or CCT7-targeting siRNAs. Representative images (right) and quantitative analyses (left) are shown (*n* = 5). (**G**) Wound-healing assays of CL1-5 and H1975 cells following CCT7 knockdown. Representative images and quantification of wound closure at the indicated time points are shown (*n* = 5). All data are presented as mean ± SD and were analyzed using Student’s *t*-test. * *p* < 0.05, ** *p* < 0.01 and *** *p* < 0.001 by Student’s *t*-test.

To investigate the clinical relevance of the CCT (chaperonin-containing TCP-1) complex in LUAD, we first analyzed the association between individual subunits and patient survival outcomes. Forest plot analysis revealed that several CCT family members were significantly associated with overall survival (OS) and relapse-free survival (RFS). Among them, CCT2 showed the strongest association with poor overall survival (Log-rank *p* = 6.4 × 10^−5^), whereas CCT7 ranked second (Log-rank *p* = 5.2 × 10^−4^) and displayed the strongest association with disease-free survival (Log-rank *p* = 3.9 × 10^−5^) compared with other CCT subunits (Figure 1A). Comparative analysis of the TCGA-LUAD dataset revealed that CCT7 expression was consistently upregulated in tumor tissues (*n* = 524) compared to adjacent normal lung tissues (*n* = 59) (Figure 1B). Consistent with the TCGA results, analyses of two independent GEO cohorts (GSE2514 and GSE32863) demonstrated significantly increased CCT7 expression in lung adenocarcinoma relative to adjacent normal tissues (GSE2514, *p* = 0.0034; GSE32863, *p* < 0.0001) (Figure 1C). We next examined expression patterns across tumor stages. Violin plot analysis revealed that CCT7 expression exhibited a marked elevation with progression, rising from stage I to stage III and stage IV. This difference was statistically significant (F = 9.76, Pr(>F) = 2.96 × 10^−6^) (Figure 1D).

To functionally characterize the role of CCT7, we silenced CCT7 expression in lung cancer cell lines CL1-5, H1299 and H1975 using small interfering RNA (siRNA) (Figure S1A). CCT7 knockdown significantly suppressed cell proliferation in H1299 and CL1-5 cells over a 3-day time-course growth assay (Figure 1E). Consistently, transwell migration assays demonstrated that CCT7 depletion markedly impaired the migratory capacity of lung cancer cells, as evidenced by a significant reduction in migrated cells compared with control cells (Figure 1F). Wound-healing assays further confirmed that CCT7 knockdown delayed wound closure, indicating suppressed migratory behavior in both CL1-5 and H1975 cells (Figure 1G). Cisplatin-based chemotherapy is a cornerstone of treatment for lung cancer and exerts its anti-tumor effects primarily by inducing DNA damage and apoptosis. To determine whether CCT7 affects the sensitivity of lung cancer cells to cisplatin-induced apoptosis, H1299 and CL1-5 cells transfected with siCtrl or siCCT7 were treated with cisplatin (30 μM) for 24 h, followed by Annexin V/PI staining and flow cytometric analysis. In H1299 cells, the percentage of apoptotic cells increased from 5.5% in siCtrl cells to 10.8% following CCT7 knockdown. A similar trend was observed in CL1-5 cells, where apoptosis increased from 4.1% in control cells to 40.5% in siCCT7-treated cells (Figure S1B). These findings indicate that loss of CCT7 enhances cisplatin-induced apoptosis in lung cancer cells, suggesting a potential role of CCT7 in promoting cell survival under chemotherapeutic stress. To further investigate whether CCT7 depletion promotes apoptotic signaling, the expression of apoptosis-related proteins was examined by Western blot analysis in H1299 and CL1-5 cells transfected with siCtrl or siCCT7. Knockdown of CCT7 resulted in a slight increase in PARP1 cleavage and a marked elevation of cleaved caspase-3 compared with control cells (Figure S1C). In parallel, the anti-apoptotic protein BCL-2 was substantially reduced following CCT7 silencing.

Collectively, these findings indicate that CCT7 is frequently upregulated in lung adenocarcinoma and plays a critical role in promoting tumor cell proliferation, migration, and survival, while its depletion enhances apoptotic signaling and sensitizes lung cancer cells to cisplatin-induced apoptosis.

### 3.2. CCT7 Silencing Activates Immune and Inflammatory Signaling Pathways

**Figure 2:**
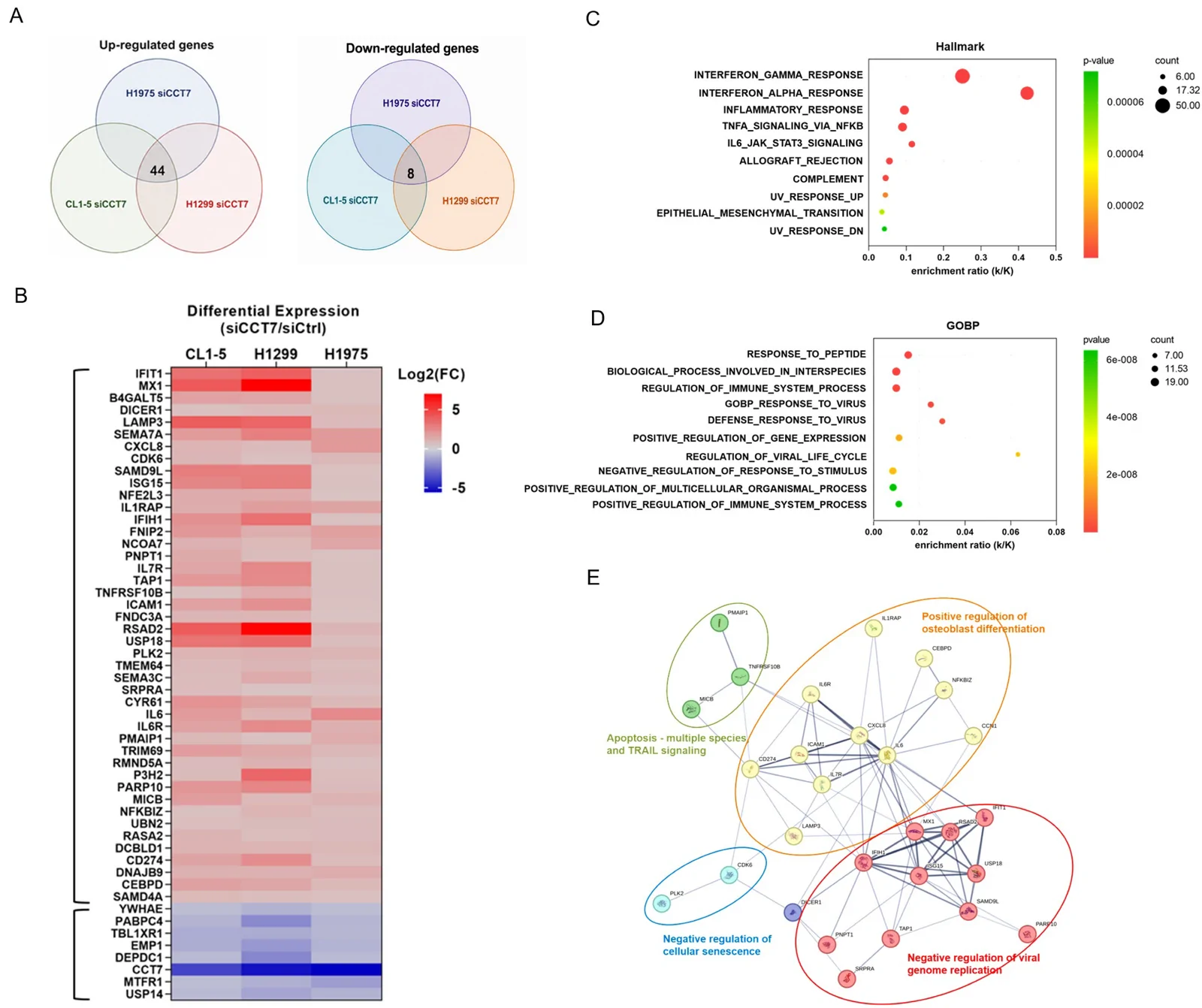
Transcriptomic and network analysis of CCT7 knockdown in lung cancer cell lines. (**A**) Venn diagrams depicting the number of commonly upregulated and downregulated genes in H1299, CL1-5 and H1975 cells after transfection with siCCT7, compared with siCtrl, as determined by RNA-seq analysis. (**B**) Heatmap showing the expression profiles of the 44 upregulated genes and 8 downregulated genes following CCT7 knockdown in H1299, CL1-5, and H1975 cells. (**C**,**D**) Hallmark and GO biological process (BP) pathway analysis performed on the shared differentially expressed genes, showing the distribution of enriched functional categories. (**E**) STRING protein–protein interaction (PPI) network analysis of the 44 commonly upregulated genes following CCT7 knockdown. The interaction network was generated using the STRING database and clustered by the Markov Cluster Algorithm (MCL).

To elucidate the molecular consequences of CCT7 depletion in LUAD, differential gene expression analysis was conducted in H1299, CL1-5, and H1975 cells following siCCT7 transfection compared with siCtrl (*p* < 0.05, |log_2_FC| ≥ 0.58). Venn analysis identified 44 genes that were consistently upregulated across all three cell lines, whereas only 8 genes were consistently downregulated (Figure 2A). The expression patterns of the 44 commonly upregulated genes across H1299, CL1-5, and H1975 cells were visualized by heatmap analysis (Figure 2B). To characterize the functional significance of transcriptional changes induced by CCT7 depletion, we performed gene set enrichment analysis (GSEA), and the top ten significantly enriched pathways were selected for visualization. Hallmark gene set enrichment analysis showed that CCT7 silencing significantly enriched immune- and inflammation-associated signatures, including interferon-α/γ response and TNFα signaling (Figure 2C). Consistently, Gene Ontology Biological Process (GOBP) analysis further confirmed significant enrichment of immune- and virus response-related pathways among genes upregulated upon CCT7 silencing (Figure 2D). Notably, the Hallmark Interferon-γ Response gene set and the GOBP immune response biological process were both significantly enriched following CCT7 silencing. These findings suggested that CCT7 may participate in the regulation of tumor immune responses, prompting us to further investigate its role in tumor immune evasion. Furthermore, STRING protein–protein interaction analysis combined with MCL clustering revealed four highly interconnected functional modules among the 44 commonly upregulated genes (Figure 2E). These clusters were primarily associated with regulation of viral genome replication, cytokine-mediated inflammatory signaling, senescence and apoptosis-related biological processes, indicating that CCT7 depletion elicits a coordinated transcriptional response involving immune-related pathways. These findings further support the conclusion that CCT7 depletion induces a coordinated immune- and stress-responsive transcriptional program in LUAD cells.

### 3.3. CCT7 Knockdown in Lung Cancer Cells Impairs Immune Responses by Increasing PD-L1 Expression

Hallmark and Gene Ontology Biological Process (GOBP) enrichment analyses revealed significant enrichment of interferon signaling and immune-related pathways following CCT7 silencing (Figure 2C,D), suggesting that CCT7 may participate in the regulation of tumor–immune interactions. To functionally validate this hypothesis, we next evaluated the impact of CCT7 depletion on T-cell activation using a tumor cell–Jurkat T-cell co-culture system.

**Figure 3:**
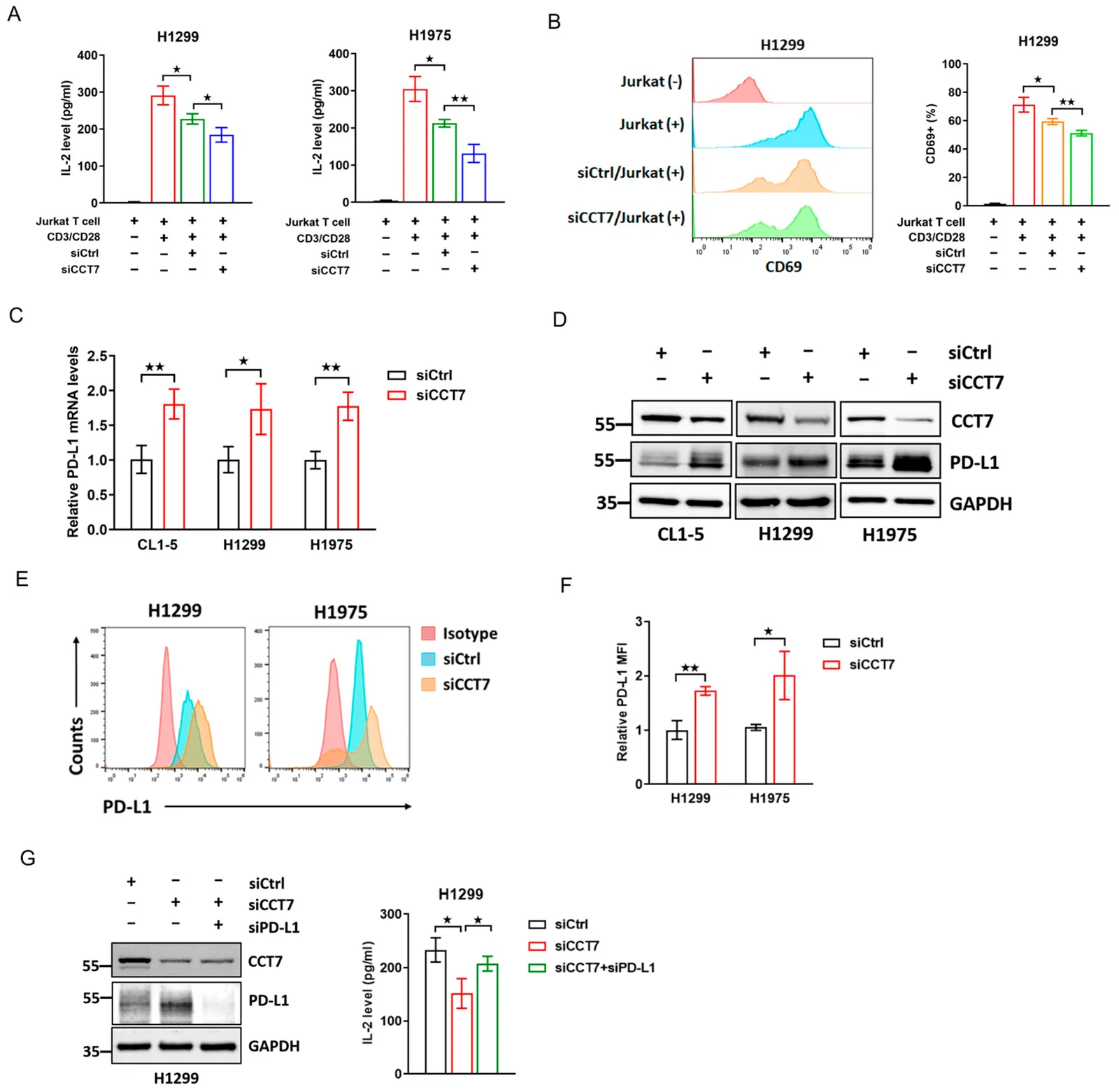
Effects of CCT7 silencing on PD-L1 expression in lung cancer cells. (**A**) Activated Jurkat T cells were co-cultured with H1299 or H1975 cells transfected with control siRNA (siCtrl) or siCCT7 for 48 h. IL-2 levels in the co-culture supernatants were quantified by ELISA (*n* = 5). (**B**) Activated Jurkat T cells were co-cultured with H1299 cells transfected with control siRNA (siCtrl) or CCT7 siRNA (siCCT7) for 24 h. CD69 expression on Jurkat T cells was analyzed by flow cytometry. Representative plots and quantification of CD69^+^ cells are shown (*n* = 3). (**C**) Quantitative real-time PCR (qPCR) analysis of PD-L1 mRNA levels in CL1-5, H1299, and H1975 cells transfected with siCCT7 compared with siCtrl. (**D**) Immunoblot analysis of PD-L1 expression in CL1-5, H1299, and H1975 cells transfected with siCtrl or CCT7 siRNA. (**E**) Flow cytometry histograms showing PD-L1 surface expression in H1299 and H1975 cells transfected with siCtrl or siCCT7, compared with isotype controls. (**F**) The relative mean fluorescence intensity (MFI) of PD-L1 on the cell surface of H1299 and H1975 cells transfected with siCtrl or siCCT7, as determined by flow cytometry. (**G**) Western blot analysis showing CCT7 and PD-L1 protein levels in H1299 and H1975 cells transfected with siCtrl, siCCT7, or siCCT7 plus siPD-L1 (left). Corresponding ELISA quantification of IL-2 levels in the culture supernatants of the same cells (right) (*n* = 5). All data are presented as mean ± SD and were analyzed using Student’s *t*-test. * *p* < 0.05; ** *p* < 0.01.

IL-2 is a pivotal cytokine produced primarily by activated T cells. CCT7 depletion markedly reduced IL-2 production compared with siCtrl-treated cells (Figure 3A). Additionally, CCT7 silencing in tumor cells significantly diminished CD69 expression in activated Jurkat T cells following 24 h of co-culture (Figure 3B). To identify candidate genes mediating the immunosuppressive effects of CCT7 depletion, we found that CD274 (PD-L1) was present within this overlapping gene set (Figure 2B). To validate whether PD-L1 expression is regulated by CCT7, qPCR was performed to evaluate the impact of CCT7 knockdown; silencing of CCT7 markedly elevated PD-L1 transcript levels in all three cell lines relative to the siCtrl group (Figure 3C). Consistently, CCT7 silencing increased PD-L1 protein expression (Figure 3D). Flow cytometric analysis further demonstrated that CCT7 knockdown significantly increased both PD-L1 surface expression (Figure 3E) and mean fluorescence intensity (MFI) (Figure 3F) compared with siCtrl-treated cells. To determine whether PD-L1 mediates the immunosuppressive effects of CCT7 depletion, PD-L1 was further silenced in CCT7-knockdown lung cancer cells. PD-L1 knockdown significantly restored IL-2 production by Jurkat T cells co-cultured with CCT7-depleted lung cancer cells (Figure 3G). These findings indicate that PD-L1 is a key mediator of the impaired T-cell activation induced by CCT7 depletion.

### 3.4. CCT7 Negatively Regulates PD-L1 Expression Through the JNK/c-Jun Pathway

**Figure 4:**
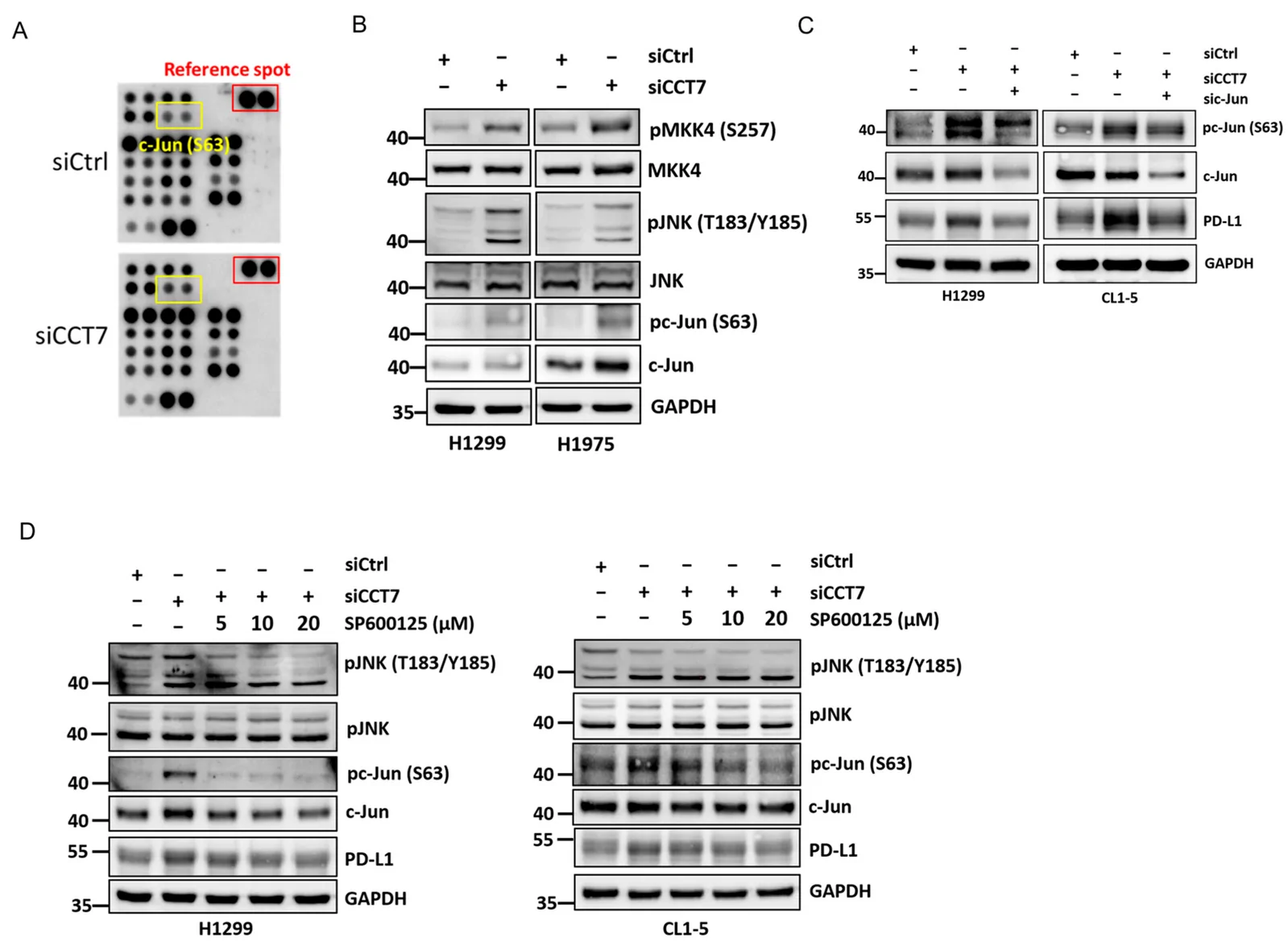
CCT7 modulates JNK/c-Jun signaling. (**A**) Human phospho-kinase arrays performed on H1975 cells transfected with siCtrl (upper panel) or siCCT7 (bottom panel). Boxed spots indicate signals for c-Jun (S63) and the reference control. (**B**) Representative validation immunoblots of the indicated lysates probing pMKK4 (S257), p-JNK (T183/Y185), p-c-Jun (S63), total MKK4, c-Jun and JNK. (**C**) Immunoblot analysis of c-Jun phosphorylation and PD-L1 expression in H1299 and CL1-5 cells transfected with siCtrl, siCCT7, or siCCT7 combined with c-Jun knockdown. (**D**) H1299 and CL1-5 cells were transfected with siCtrl or siCCT7, followed by dose-dependent treatment with the JNK inhibitor SP600125 (5–20 µM) for 24 h. Whole-cell lysates were subjected to immunoblotting with the indicated antibodies.

To delineate the signaling pathways through which CCT7 regulates PD-L1 expression, we performed a phospho-kinase array to screen for alterations in multiple canonical pathways known to control PD-L1 transcription. The array revealed increased phosphorylation signals for c-Jun at Ser63 following CCT7 depletion in H1975 cells (Figure 4A).

Phosphorylation of JNK at Thr183/Tyr185 is a hallmark of JNK activation, whereas phosphorylation of c-Jun at Ser63, a major downstream target of JNK, enhances its transcriptional activity. To validate the phospho-kinase array results, we examined the phosphorylation status of c-Jun and its upstream kinases, MKK4 and JNK, in lung cancer cells. CCT7 knockdown markedly increased the phosphorylation of c-Jun (S63), accompanied by increased phosphorylation of the upstream kinases MKK4 and JNK (Figure 4B). These findings indicate that CCT7 depletion activates the MKK4–JNK–c-Jun signaling axis. To determine whether CCT7-mediated PD-L1 upregulation depends on JNK/c-Jun signaling, H1299 and CL1-5 cells transfected with siCCT7 or siCtrl were treated with c-Jun siRNA or the JNK inhibitor SP600125 (5–20 µM) for 24 h. Both genetic and pharmacological inhibition of JNK/c-Jun signaling markedly attenuated PD-L1 protein expression under CCT7-silenced conditions (Figure 4C,D), indicating that CCT7 regulates PD-L1 expression through a JNK/c-Jun-dependent mechanism.

### 3.5. CCT7 Silencing Activates JNK/c-Jun Signaling via DR5 Induction

**Figure 5:**
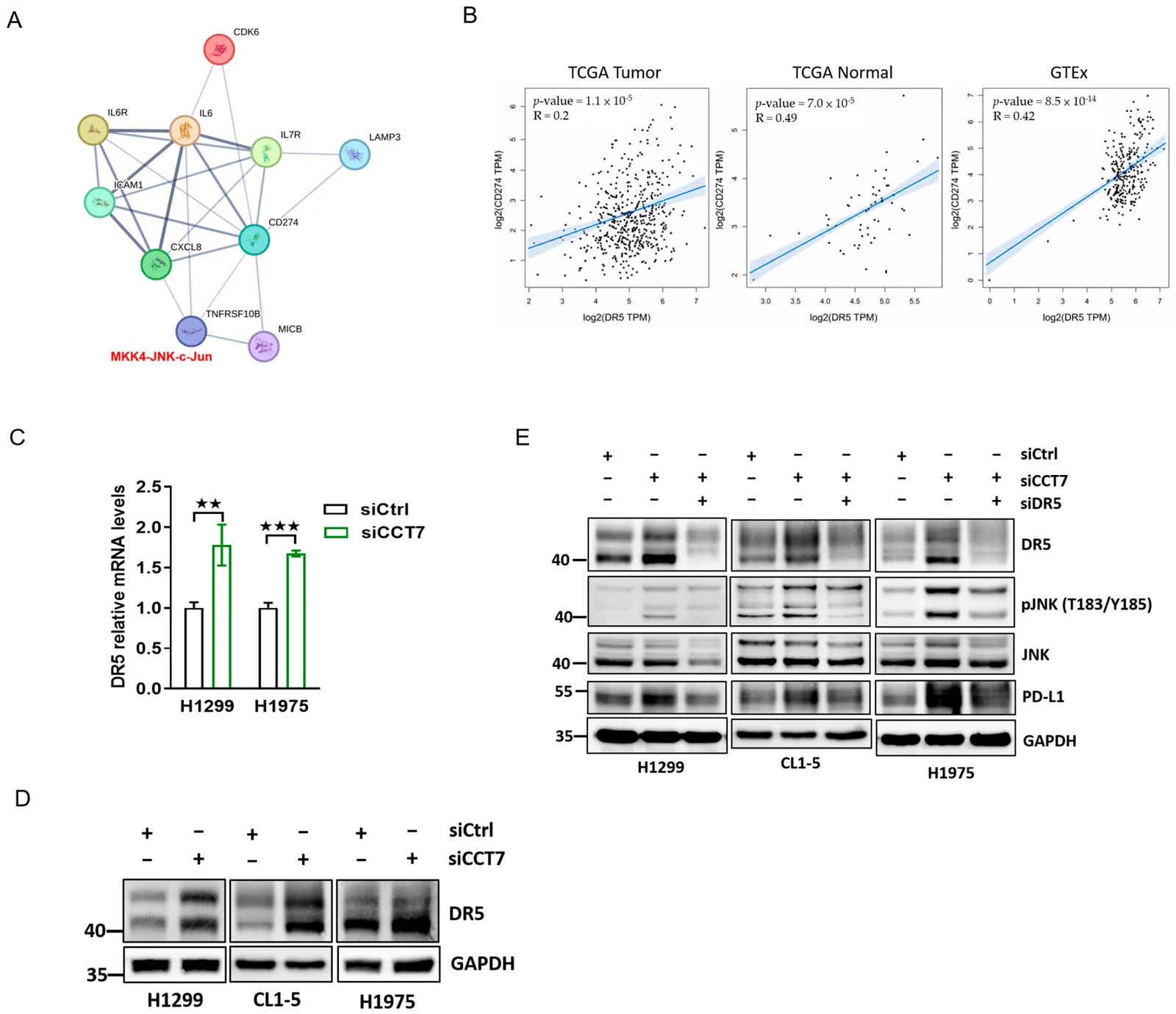
DR5 mediates CCT7 depletion-induced activation of the JNK/PD-L1 signaling pathway. (**A**) STRING protein–protein interaction network highlighting TNFRSF10B (DR5) and its interaction partners. (**B**) Correlation between PD-L1 and DR5 expression in TCGA LUAD tumors, adjacent normal tissues, and GTEx normal lung tissues. R and corresponding *p* values are shown. (**C**) qRT-PCR analysis of TNFRSF10B (DR5) mRNA expression following CCT7 knockdown in H1299 and H1975 cells. (**D**) Immunoblot analysis of DR5 protein expression in H1299, H1975, and CL1-5 cells following transfection with control siCtrl or CCT7 siRNA. (**E**) Immunoblot analysis of DR5, p-JNK (T183/Y185), total JNK, and PD-L1 expression in lung cancer cell lines transfected with siCtrl or siCCT7, with or without DR5 knockdown. All data are presented as mean ± SD and were analyzed using Student’s *t*-test. ** *p* < 0.01; *** *p* < 0.001.

To identify candidate mediators linking CCT7 depletion to JNK/c-Jun activation, we systematically screened the commonly upregulated genes following CCT7 knockdown. STRING network analysis revealed that TNFRSF10B (DR5) was functionally connected with CD274 (PD-L1) and other immune-related molecules (Figure 5A). Notably, among the identified candidates, DR5 was the only gene with a reported ability to activate the MKK4–JNK–c-Jun signaling cascade. Given its unique association with both JNK/c-Jun signaling and the PD-L1-centered immune regulatory network, DR5 was selected for subsequent mechanistic investigation. To further explore the relationship between DR5 and PD-L1, correlation analyses were performed using public transcriptomic datasets. TNFRSF10B expression was positively correlated with CD274 expression in TCGA LUAD tumors (R = 0.20, *p* = 1.1 × 10^−5^), adjacent normal lung tissues (R = 0.49, *p* = 7 × 10^−5^), and GTEx normal lung tissues (R = 0.42, *p* = 8.5 × 10^−14^) (Figure 5B). These findings suggest a consistent association between TNFRSF10B and CD274 expression in both normal lung tissues and LUAD.

To determine whether CCT7 regulates DR5 expression, we examined DR5 mRNA and protein levels following CCT7 knockdown in lung adenocarcinoma cell lines. Silencing CCT7 significantly increased DR5 mRNA and protein expression levels in both H1299 and H1975 cells compared with siCtrl-transfected cells (Figure 5C,D). We further examined whether DR5 upregulation following CCT7 silencing is associated with enhanced JNK phosphorylation and increased PD-L1 expression. As expected, silencing of DR5 attenuated siCCT7-induced JNK phosphorylation and reduced PD-L1 expression across multiple lung cancer cell lines (Figure 5E), suggesting that DR5 is required for activation of the JNK pathway and downstream PD-L1 regulation in the context of CCT7 deficiency.

### 3.6. CCT2 Depletion Suppresses the DR5–JNK–c-Jun–PD-L1 Axis

Previous studies have suggested that the expression of CCT subunits is mutually regulated, implying potential functional interplay within the CCT complex [30,31]. To determine whether other CCT members regulate the DR5–PD-L1 axis similarly to CCT7, we analyzed expression correlations among CCT family genes using the TCGA LUAD dataset together with proteomic profiling data. Notably, among all CCT subunits, CCT2 exhibited the weakest correlation with CCT7 in the TCGA LUAD dataset (Pearson’s R = 0.160), consistent with the minimal effect of CCT7 knockdown on CCT2 protein expression (siCCT7/siCtrl = 0.88) (Figure S2A,B). To determine whether CCT7 and CCT2 regulate each other’s expression, we examined their protein levels following siRNA-mediated knockdown. Silencing CCT7 did not alter CCT2 expression, and vice versa (Figure S2C,D). These results indicate that CCT7 and CCT2 are regulated independently at the protein level, providing a rationale to directly compare the phenotypic consequences of CCT7 and CCT2 depletion in lung cancer cells.

**Figure 6:**
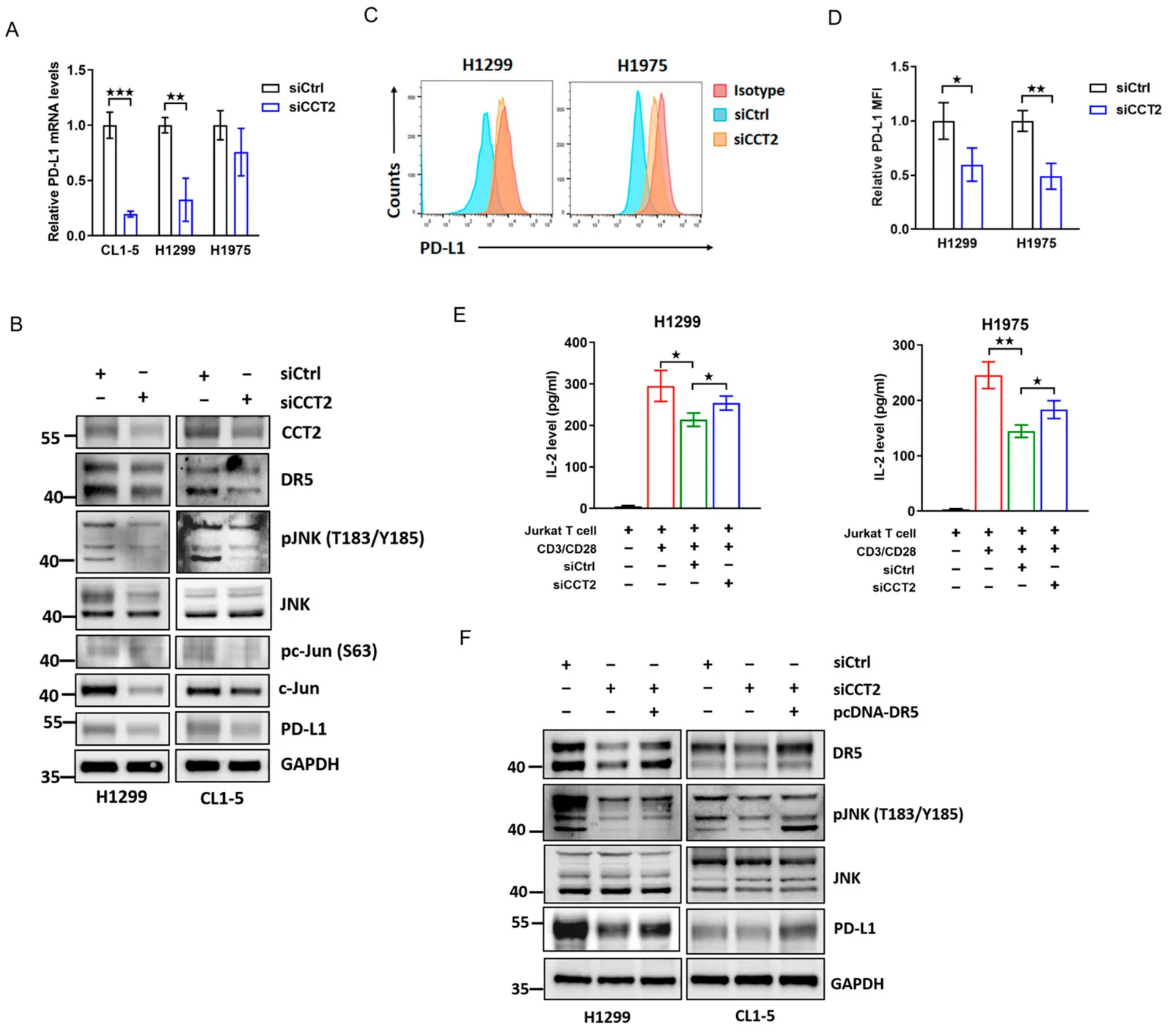
CCT2 knockdown suppresses the DR5–JNK–c-Jun–PD-L1 signaling pathway. (**A**) Quantitative RT-PCR analysis of PD-L1 mRNA expression following siRNA-mediated CCT2 knockdown in CL1-5, H1299, and H1975 cells. (**B**) Representative immunoblot analysis of CCT2, DR5, p-JNK (T183/Y185), total JNK, p-c-Jun (S63), total c-Jun, and PD-L1 in H1299 and CL1-5 cells following CCT2 knockdown. (**C**) Representative flow cytometry histograms showing cell surface PD-L1 expression in H1299 and H1975 cells following CCT2 knockdown (*n* = 3). (**D**) Quantification of relative PD-L1 mean fluorescence intensity (MFI) (*n* = 3). (**E**) IL-2 secretion by activated Jurkat T cells following co-culture with H1299 or H1975 cells transfected with the indicated siRNAs (*n* = 5). (**F**) Representative immunoblot analysis of DR5, p-JNK (T183/Y185), total JNK, and PD-L1 in H1299 and CL1-5 cells transfected with the indicated siRNAs or a DR5 expression plasmid. All data are presented as mean ± SD and were analyzed using Student’s *t*-test. * *p* < 0.05; ** *p* < 0.01; *** *p* < 0.001.

In contrast to CCT7 depletion, CCT2 knockdown elicited an opposite pattern of signaling and immune-related responses in lung cancer cells. Silencing CCT2 reduced PD-L1 mRNA expression (Figure 6A), accompanied by decreased phosphorylation of JNK and c-Jun, as well as reduced PD-L1 protein expression in both H1299 and CL1-5 cells (Figure 6B). Consistently, flow cytometric analysis demonstrated a significant reduction in both cell surface PD-L1 expression and mean fluorescence intensity (MFI) (Figure 6C,D). Functionally, co-culture of siCCT2-transfected tumor cells with activated Jurkat T cells significantly increased IL-2 production compared with siCtrl-treated cells in both H1299 and H1975 cells (Figure 6E), indicating enhanced T cell activation following CCT2 depletion. To determine whether DR5 mediates the regulation of PD-L1 by CCT2, DR5 was ectopically expressed in siCCT2-treated cells, which effectively restored JNK phosphorylation and PD-L1 expression (Figure 6F). Collectively, these findings indicate that CCT2 and CCT7 converge on the same DR5–JNK–PD-L1 signaling axis but regulate this pathway in opposite directions.

## 4. Discussion

Programmed death-ligand 1 (PD-L1) plays a central role in immune evasion in lung cancer by suppressing T cell activation and effector function through engagement of the PD-1 receptor. In lung adenocarcinoma, elevated PD-L1 expression has been associated with advanced disease stage, poor prognosis, and resistance to anti-tumor immune responses [32,33]. Mechanistically, PD-L1 expression in lung cancer cells is regulated by multiple oncogenic and stress-responsive signaling pathways, including MAPK, JAK/STAT, AKT/mTOR and NF-κB signaling, as well as cellular stress and inflammatory cues within the tumor microenvironment [34,35,36]. In this context, understanding how tumor-intrinsic factors regulate PD-L1 expression is critical for elucidating mechanisms of immune escape and therapeutic resistance in lung adenocarcinoma.

In this study, we identify CCT7 as a critical regulator of tumor progression and immune modulation in lung adenocarcinoma and further reveal a functional divergence between CCT7 and another TRiC/CCT subunit, CCT2. Although both proteins belong to the same chaperonin complex, our integrative analyses demonstrate that CCT7 and CCT2 exhibit low correlation at the transcript and protein levels and are regulated independently in lung cancer cells. Importantly, depletion of CCT7 and CCT2 elicited opposing effects on DR5–JNK–c-Jun signaling, PD-L1 expression, and T cell-associated immune activity, underscoring subunit-specific roles of the TRiC/CCT complex beyond its canonical protein-folding function. Mechanistically, CCT7 silencing induced DR5 upregulation and activation of the MKK4–JNK–c-Jun axis, leading to increased PD-L1 expression at both the transcript and protein levels. This signaling cascade was functionally relevant, as genetic or pharmacological inhibition of JNK/c-Jun attenuated PD-L1 induction under CCT7-deficient conditions. Given that DR5 is a death receptor traditionally associated with extrinsic apoptosis, our findings extend its functional scope by linking DR5 activation to stress-responsive JNK signaling and immune checkpoint regulation. The concurrent activation of apoptotic markers, including cleaved PARP and caspase-3, suggests that CCT7 loss may impose proteotoxic or cellular stress, thereby engaging DR5-dependent signaling pathways that intersect with immune evasion mechanisms.

Functionally, CCT7 depletion suppressed IL-2 production by Jurkat T cells in co-culture assays, an effect that was largely reversed by PD-L1 knockdown, indicating that PD-L1 upregulation is a key mediator of CCT7-dependent immunosuppression. These findings highlight a dual effect of CCT7 depletion. While CCT7 depletion suppresses lung cancer cell proliferation and migration and enhances apoptosis, it simultaneously induces PD-L1 expression and suppresses T-cell activation. Thus, although CCT7 depletion exerts direct inhibitory effects on lung cancer cells, it may also trigger a compensatory immune-evasion response through PD-L1 upregulation. Whether CCT7 targeting in combination with PD-1/PD-L1 blockade could provide greater therapeutic benefit warrants further investigation. In contrast, CCT2 silencing resulted in reduced DR5 expression, attenuation of JNK/c-Jun signaling, downregulation of PD-L1, and enhanced IL-2 production by T cells. Notably, CCT2 knockdown did not alter CCT7 protein levels, and vice versa, excluding the possibility that these effects arise from global destabilization of the TRiC/CCT complex. Instead, our data support the concept that individual CCT subunits exert distinct, and even opposing, regulatory functions in tumor-immune crosstalk. This functional asymmetry highlights the complexity of chaperonin biology in cancer and cautions against viewing TRiC/CCT subunits as interchangeable components.

Collectively, our findings position CCT7 as a tumor-promoting factor that regulates immune checkpoint signaling through the DR5–JNK–c-Jun–PD-L1 axis, while identifying CCT2 as a functionally divergent subunit with immune-permissive effects. These results not only provide new insight into subunit-specific roles of the TRiC/CCT complex in lung cancer but also suggest that therapeutic targeting of CCT7 may require consideration of the compensatory PD-L1-mediated immune response induced by CCT7 depletion. Several limitations of this study should be acknowledged. First, although our findings delineate a DR5–JNK–c-Jun–PD-L1 signaling axis downstream of CCT7 silencing using in vitro lung cancer cell models and immune co-culture systems, the in vivo relevance of this pathway remains to be fully validated. Future studies employing syngeneic or humanized mouse models will be essential to determine how CCT7-dependent immune modulation influences tumor growth, immune infiltration, and response to immune checkpoint blockade in an intact tumor microenvironment. Second, while DR5 upregulation was shown to be required for JNK activation and PD-L1 induction following CCT7 depletion, the precise molecular mechanisms linking CCT7 loss to DR5 stabilization or activation remain unresolved. Stress-induced activation of ATF4 is known to promote CHOP expression, and CHOP has been reported to directly regulate DR5 transcription [37,38]. Accordingly, we examined CHOP and ATF4 mRNA levels upon CCT7 silencing. Surprisingly, both transcripts were reduced rather than upregulated, suggesting that CCT7-mediated regulation of DR5 expression occurs independently of stress-induced ATF4–CHOP signaling.

Finally, although this study highlights functional divergence among TRiC/CCT subunits by contrasting CCT7 and CCT2, whether similar immune-regulatory roles extend to other CCT family members warrants systematic evaluation. Elucidating subunit-specific dependencies may ultimately facilitate the development of more selective therapeutic strategies targeting CCT7-mediated tumor immune evasion while preserving essential chaperonin functions.

## 5. Conclusions

In conclusion, our findings identify distinct and opposing functions of TRiC/CCT subunits in regulating tumor immune evasion in LUAD. CCT7 deficiency activates the TNFRSF10B (DR5)–MKK4–JNK–c-Jun signaling cascade, leading to transcriptional upregulation of PD-L1 and suppression of T-cell activation. In contrast, CCT2 deficiency attenuates this signaling axis, resulting in reduced PD-L1 expression and enhanced T-cell activity. These findings uncover previously unrecognized functional divergence among TRiC/CCT subunits and establish the DR5–JNK–PD-L1 axis as a critical mechanism regulating immune responses, highlighting the potential therapeutic relevance of CCT7 and CCT2 in cancer immunotherapy.

## Data Availability

The original contributions presented in this study are included in the article/Supplementary Materials. Further inquiries can be directed to the corresponding authors.

## Supplementary Materials

The following supporting information can be downloaded at: https://www.mdpi.com/article/10.3390/cells15171613/s1.

## Abbreviations

The following abbreviations are used in this manuscript:

CCT	Chaperonin-containing TCP-1
LUAD	Lung adenocarcinoma
NSCLC	Non-small cell lung cancer
ICIs	Immune checkpoint inhibitors
TCGA	The Cancer Genome Atlas
ICB	Immune checkpoint blockade
TRiC	TCP-1 Ring Complex
GSEA	Gene set enrichment analysis
DEGs	Differentially expressed genes
GEO	Gene Expression Omnibus
GOBP	Gene Ontology Biological Process
