# Supplementary material for: Differential Modulation of the DR5–JNK–PD-L1 Signaling Axis by TRiC/CCT Subunits CCT7 and CCT2 Shapes Tumor Immune Evasion in Lung Adenocarcinoma
Source: Cells. 2026 Sep 4;15(17):1613. doi: 10.3390/cells15171613 (PMC13565191; doi:10.3390/cells15171613)
Supplement: Supplementary file 1 [file cells-15-01613-s001.zip › Supplementary figures.pdf]

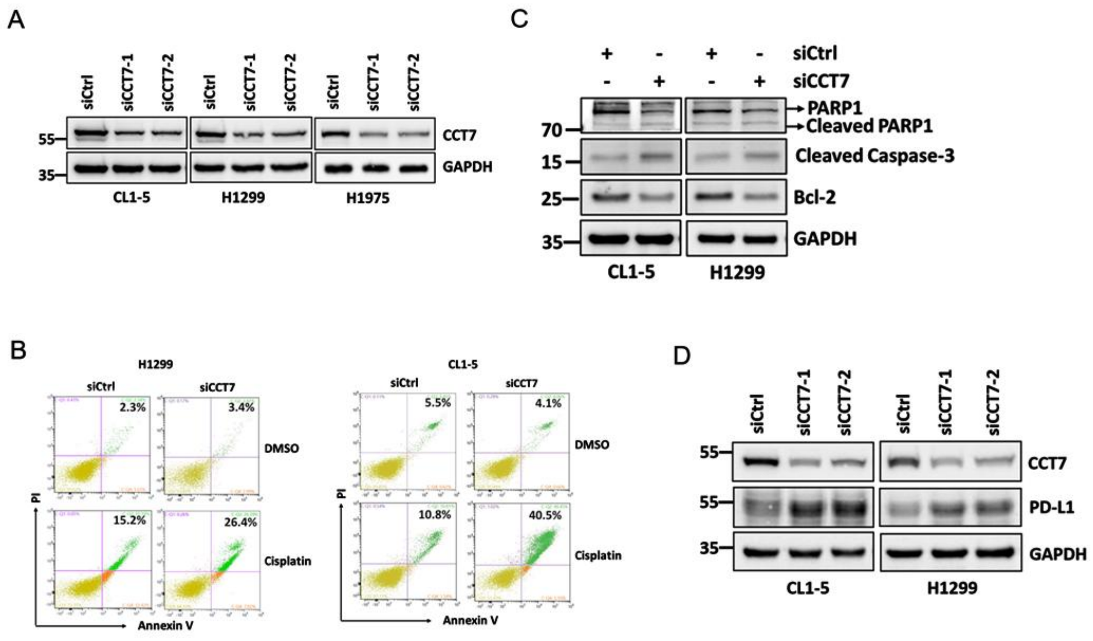

Supplementary Figure S1. (A) CL1-5, H1299, and H1975 cells were transfected with control siRNA (siCtrl) or two independent siRNAs targeting CCT7 (siCCT7-1 and siCCT7-2). Western blot analysis to examine CCT7 protein expression. GAPDH was used as an internal loading control. (B) Flow cytometric analysis of apoptosis in H1299 and CL1-5 cells transfected with siCtrl or siCCT7 24 h and then treated with or without cisplatin (30 μM) for 24 h. Cells were stained with Annexin V/PI, and the percentages of apoptotic cells are shown. (C) Immunoblot analysis of apoptosis-related markers, including cleaved PARP (PARP1), cleaved caspase-3 (Caspase-3) and BCL-2, in CL1-5 and H1299 cells following CCT7 knockdown. (D) CL1-5 and H1299 cells were transfected with two independent siRNAs targeting CCT7 (siCCT7-1 and siCCT7-2) or a non-targeting control siRNA (siCtrl). CCT7 and PD-L1 protein expression was assessed by Western blotting.

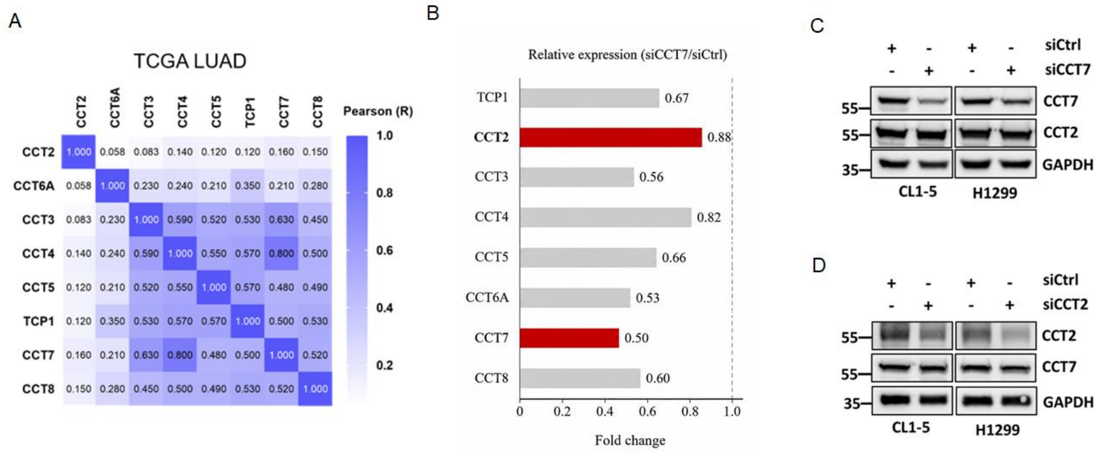

Supplementary Figure S2. (A) Heatmap of Pearson correlation coefficients (R) among CCT complex genes in the TCGA-LUAD dataset. Color intensity indicates the strength of correlation between gene expression levels. (B) Relative protein abundance of CCT complex subunits after CCT7 knockdown based on proteomic profiling (LC–MS/MS) in H1975 cells. Values indicate the siCCT7/siCtrl ratio for each CCT subunit. (C, D) Western blot analysis of the indicated proteins in lung cancer cells transfected with siCtrl or siCCT7/siCCT2 in CL1-5 and H1299 cells. Whole-cell lysates were subjected to immunoblotting with the indicated antibodies.
